# Checklist of butterflies (Insecta: Lepidoptera) from Serra do Intendente State Park - Minas Gerais, Brazil

**DOI:** 10.3897/BDJ.2.e3999

**Published:** 2014-11-25

**Authors:** Izabella Nery, Natalia Carvalho, Henrique Paprocki

**Affiliations:** † Pontifícia Universidade Católica de Minas Gerais, Belo Horizonte, Brazil

**Keywords:** Espinhaço Mountain Range, Arthropoda, frugivorous butterflies, Peixe Tolo, inventory

## Abstract

In order to contribute to the butterflies’ biodiversity knowledge at Serra do Intendente State Park - Minas Gerais, a study based on collections using Van Someren-Rydon traps and active search was performed. In this study, a total of 395 butterflies were collected, of which 327 were identified to species or morphospecies. 263 specimens were collected by the traps and 64 were collected using entomological hand-nets; 43 genera and 60 species were collected and identified.

## Introduction

The Lepidoptera is comprised of butterflies and moths; it is one of the main orders of insects which has approximately 157,424 described species (Freitas and Marini-Filho 2011, Zhang 2011). The butterflies, object of this study, have approximately 19,000 species described worldwide (Heppner 1991). The occurrence of 3,300 species is estimated for Brazil, with more than 1,600 for Minas Gerais state (Casagrande et al. 1998). The group of butterflies studied belong to the superfamilies Papilionoidea and Hesperioidea and are subdivided into six families: Hesperiidae (Hesperioidea) and Papilionidae, Pieridae, Lycaenidae, Riodionidae, and Nymphalidae (Papilionoidea) (Brown Jr. and Freitas 1999).

These insects are characterized as holometabolous, terrestrial, and diurnal. They are plant material chewers in the larval stage and liquid suckers in adulthood (Heppner 1991). Butterflies are insects that inhabit almost all terrestrial natural ecosystems (Freitas et al. 2003) and the presence of these creatures in a habitat is related to the availability of food resources and conditions such as temperature, relative humidity, and sunlight incidence (Brown Jr. and Hutchings 1997). They are divided into two guilds, according to adults’ dietary habits: the nectarivorous and those who feed on fermented fruit, excrements, and exudates of decaying plants and animals (Uehara-Prado et al. 2007).

Butterflies are important indicators of environmental quality, because they are diverse, can be easily viewed, captured, identified, and manipulated by researchers (Mielke and Casagrande 1997). They are present throughout the year, and exhibit rapid responses regarding environmental disturbances (Öckinger et al. 2006). The larvae are considered pests of agricultural crops causing important damage leading to economic loss. However, they are important pollinators in adulthood (Isehard and Romanowski 2004, Triplehorn and Johnson 2011).

There are few studies about butterfly biodiversity in the Espinhaço Biosphere Reserve (Wilson 1997). The knowledge of Lepidoptera biodiversity in Minas Gerais, is still scarce (Casagrande et al. 1998). There is no published literature about butterfly biodiversity and distribution for the Espinhaço mountain range within Minas Gerais. The number of Lepidoptera checklists for Brazil is still very small and this effort contributes to a better understanding of biodiversity distribution in the country.

This work inventories butterflies on a state conservation area called Serra do Intendente State Park (PESI), Minas Gerais, Brazil.

## Materials and methods

*Study Site*

**Figure 1.**
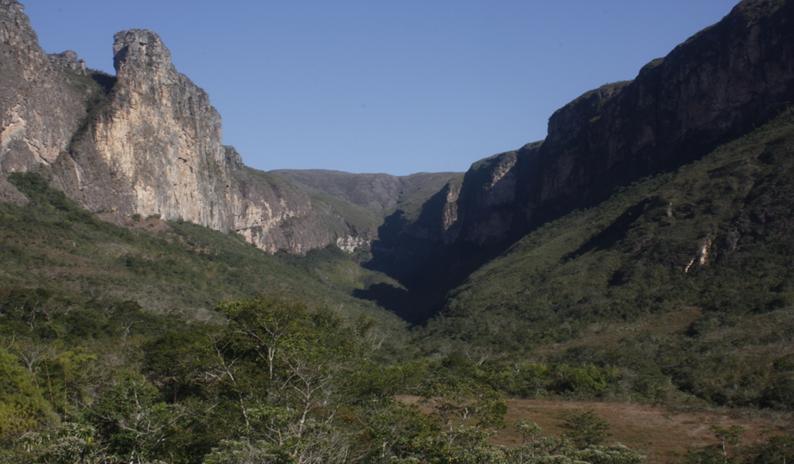
Study area - Peixe Tolo Natural Reserve and Serra do Intendente State park, Minas Gerais, Brazil.

**Figure 2.**
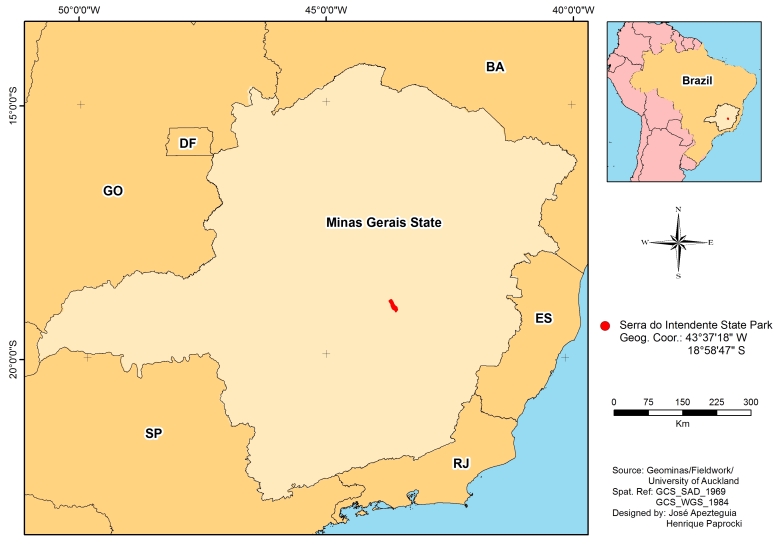
Location map of Serra do Intendente State Park, Minas Gerais, Brazil.

The study was conducted in the region of Serra do Espinhaço, more precisely, within the Serra do Intendente State Park (Fig. 2) and the Peixe Tolo Natural Reserve, Minas Gerais (Fig. 1). The park features approximately 13,508 ha and is an important area for Espinhaço mountains biodiversity conservation.

The climate is mesothermal, characterized by mild, humid summers and dry, cold winters. The average annual rainfall is 1,600 mm. The annual mean temperature is 18, 7ºC (Araujo et al. 2005, Silva et al. 2009). The predominant topography is the mountainous escarpment, mixed with rocky outcrops. The vegetation is mosaic and it is characterized by the presence of striking landscapes of three biomes: Atlantic Forest, Caatinga, and Cerrado (Andrade and Domingues 2013).

*Data Collection*

**Figure 3.**
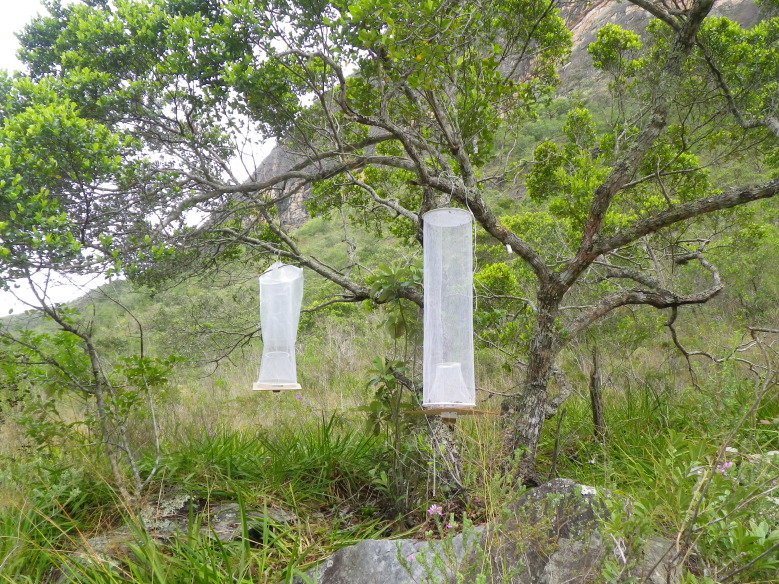
Photographie of sampling point with replica traps in Serra do Intendente State park, Minas Gerais, Brazil.

The collections began in April 2012 and were completed in February of 2013. During this period four collections (two in the rainy season and two in the dry season) were performed. Each collection was performed for five days. The study area was divided into two areas throughout the Peixe Tolo River basin and in each area, forty Van Someren-Rydon traps were distributed. Twenty traps were located on the right bank and other twenty on the left bank of the Peixe Tolo River (Fig. 3). In these traps, baits made of a mixture of ripe banana and sugarcane syrup was used; the solution was left fermenting for forty-eight hours before exposure in the traps.

Throughout the collection period, active search of butterflies was performed in order to capture non-frugivorous butterflies. The specimens collections were conducted throughout the day, starting around 10am until 3pm. The butterflies captured were immediately killed through abdomen compression in order to avoid damage that could compromise identification.

*Data analysis*

The collected material was mounted, identified and labeled in the PUC Minas Natural Sciences Museum entomological collection laboratory. The identification of the individuals was made using Devries (1997), Freitas et al. (2003), D'Abrera (1987b), D'Abrera (1987a), D'Abrera (1988) and the website Butterflies of America (accessible at http://butterfliesofamerica.com/). Genera and species were confirmed by Dr. André Freitas, from the Department of Animal Biology, Universidade Estadual de Campinas. Furthermore, a comparison with available identified species in the Lepidoptera collection in the Invertebrates Laboratory (PUC Minas) was performed.

## Data resources

In this study 394 individuals were captured, and 327 were identified. Sixty-seven individuals were not identified to genus or species due to bad specimen conditions or incipient systematics.

The families represented in this study were: Nymphalidae, Pieridae, Hesperiidae, Lycaenidae, Papilionidae and Riodinidae. A total of 299 individuals belonging to the Nymphalidae, 15 from the Pieridae, four from the Hesperiidae, four from the Lycaenidae, three from the Papilionidae, and one species from the Riodinidae.

A total of 263 butterflies were collected in traps and 63 using entomological hand-nets. The collections gathered specimens belonging to 43 genera and 60 species (Table 1). During the rainy seasons 181 individuals were collected in which 177 were collected by traps and 4 by entomological hand-nets (Table 2). In the dry seasons 145 individuals were collected: of those, 86 were collected in traps and 59 by entomological hand-nets (Table 3).

## Checklists

### Checklist of butterflies (Insecta: Lepidoptera) from Serra do Intendente State Park - Minas Gerais, Brazil

#### Adelpha pleasure

(Hübner, 1823)

http://eol.org/pages/4090956/overview

#### Adelotypa malca

(Shaus, 1902)

http://www.butterfliesofamerica.com/L/adelotypa_malca.htm

#### Archaeoprepona demophon

(Linnaus, 1758)

http://eol.org/pages/168926/overview

http://butterfliesofamerica.com/L/archaeoprepona_d_demophoon_types.htm

#### Autochton zarex

(Hübner,1818)

http://eol.org/pages/181967/overview

#### Anartia amathea

(Linnaus, 1758)

http://eol.org/pages/159066/overview

http://butterfliesofamerica.com/anartia_a_amathea_types.htm

#### Ascia monuste

(Linnaus, 1764)

http://eol.org/pages/172859/overview

http://www.butterfliesofamerica.com/L/ascia_m_monuste_types.htm

#### Blepolenis batea

(Hübner, 1821)

http://eol.org/pages/11555451/overview

http://www.butterfliesofamerica.com/L/blepolenis_b_batea_types.htm

#### Caligo arisbe

Hübner, 1820

http://eol.org/pages/149491/overview

http://www.butterfliesofamerica.com/L/caligo_a_arisbe_types.htm

#### Callicore sorana

(Godart, 1832)

http://eol.org/pages/4090003/overview

http://www.butterfliesofamerica.com/L/callicore_s_sorana_types.htm

#### Catonephele acontius

(Linnaus, 1771)

http://eol.org/pages/163630/overview

http://www.butterfliesofamerica.com/L/catonephele_a_acontius_types.htm

#### Colobura dirce

(Linnaus, 1764)

http://eol.org/pages/156101/overview

http://www.butterfliesofamerica.com/L/colobura_d_dirce_types.htm

#### Dryas iulia

(Fabricius, 1775)

http://eol.org/pages/158533/overview

http://www.butterfliesofamerica.com/L/dryas_i_iulia_types.htm

#### Euptychoides castrensis

(Shaus, 1902)

http://eol.org/pages/12083351/overview

http://www.butterfliesofamerica.com/L/euptychoides_castrensis_types.htm

#### Eryphanis reevesii

(Doubleday, 1849)

http://eol.org/pages/146531/overview

http://www.butterfliesofamerica.com/L/eryphanis_r_reevesii_types.htm

#### Eresia lansdorfi

(Godart, 1819)

http://eol.org/pages/160030/overview

http://www.butterfliesofamerica.com/L/eresia_lansdorfi_types.htm

#### Eurema albula

(Cramer, 1775)

http://eol.org/pages/176703/overview

http://butterfliesofamerica.com/L/eurema_a_albula_types.htm

#### Eurema elathea

(Cramer, 1777)

http://eol.org/pages/178177/overview

http://www.butterfliesofamerica.com/L/eurema_e_elathea.htm

#### Eurema phiale

(Cramer, 1775)

http://eol.org/pages/184116/overview

http://www.butterfliesofamerica.com/L/eurema_p_phiale_types.htm

#### Eurema sp.

http://eol.org/pages/19949/overview

#### Godartiana muscosa

(Butler, 1870)

http://eol.org/pages/961111/overview

http://www.butterfliesofamerica.com/L/godartiana_muscosa_types.htm

#### Hamadryas amphinome

(Linnaus, 1767)

http://eol.org/pages/166283/overview

http://www.butterfliesofamerica.com/L/hamadryas_a_amphinome_types.htm

#### Hamadryas februa

(Hübner, 1816/24)

http://eol.org/pages/166346/overview

http://www.butterfliesofamerica.com/L/hamadryas_f_februa_types.htm

#### Hamadryas feronia

(Linnaus, 1758)

http://eol.org/pages/166361/overview

http://www.butterfliesofamerica.com/L/hamadryas_f_feronia_types.htm

#### Heliconius besckei

(E. Ménétriés, 1857)

http://eol.org/pages/155098/overview

http://www.butterfliesofamerica.com/L/heliconius_besckei_types.htm

#### Heliconius erato

(Linnaus, 1764)

http://eol.org/pages/151378/overview

http://www.butterfliesofamerica.com/L/heliconius_e_erato_types1.htm

#### Heliconius ethilla

(Godart, 1819)

http://eol.org/pages/157369/overview

http://www.butterfliesofamerica.com/L/heliconius_e_ethilla_types.htm

#### Heliopetes omrina

(Butler, 1870)

http://eol.org/pages/185550/overview

http://www.butterfliesofamerica.com/L/heliopetes_omrina_types.htm

#### Junonia evarete

(Cramer, 1782)

http://eol.org/pages/162840/overview

http://www.butterfliesofamerica.com/junonia_e_evarete_types.htm

#### Junonia genoveva

(Cramer, 1782)

http://eol.org/pages/157257/overview

http://www.butterfliesofamerica.com/junonia_g_genoveva_types.htm

#### Leptotes cassius

(Cramer, 1775)

http://eol.org/pages/264320/overview

http://www.butterfliesofamerica.com/L/leptotes_c_cassius_types.htm

#### Leptotes sp.

http://eol.org/pages/33170/overview

#### Marpesia chiron

(Fabricius, 1775)

http://eol.org/pages/165801/overview

http://www.butterfliesofamerica.com/L/marpesia_c_chiron_types.htm

#### Memphis moruus

(Fabricius, 1775)

http://eol.org/pages/29501563/overview

http://www.butterfliesofamerica.com/L/memphis_m_moruus_types.htm

#### Memphis otrere

(Hübner, 1825)

http://eol.org/pages/29514890/overview

http://www.butterfliesofamerica.com/L/memphis_otrere_types.htm

#### Memphis ryphea

(Geyer, 1834)

http://eol.org/pages/23311886/overview

#### Memphis sp.

http://eol.org/pages/19988/overview

#### Morpho helenor

(Cramer, 1782)

http://eol.org/pages/138539/overview

http://www.butterfliesofamerica.com/L/morpho_h_helenor_types.htm

#### Narope cyllarus

(Westwood, 1851)

http://eol.org/pages/148144/overview

http://www.butterfliesofamerica.com/L/narope_cyllarus_types.htm

#### Opsiphanes cassiae

(Linnaus, 1758)

http://eol.org/pages/150133/overview

http://www.butterfliesofamerica.com/L/opsiphanes_c_cassiae_types.htm

#### Opsiphanes quiteria

(Stoll, 1782)

http://eol.org/pages/147972/overview

http://www.butterfliesofamerica.com/L/opsiphanes_q_quiteria_types.htm

#### Opoptera syme

(Hübner, 1822/26)

http://eol.org/pages/148836/overview

http://www.butterfliesofamerica.com/L/opoptera_syme_types.htm

#### Pareuptychia ocirrhoe

(Fabricius, 1777)

http://eol.org/pages/138517/overview

http://www.butterfliesofamerica.com/L/pareuptychia_o_ocirrhoe.htm

#### Paryphthimoides undulata

(Butler, 1867)

http://www.butterfliesofamerica.com/L/paryphthimoides_undulata_types.htm

#### Prepona laertes

(Hübner, 1811)

http://eol.org/pages/168780/overview

http://www.butterfliesofamerica.com/L/prepona_l_laertes_types.htm

#### Pseudolycaena marsyas

(Linnaus, 1758)

http://eol.org/pages/261603/overview

http://www.butterfliesofamerica.com/L/pseudolycaena_marsyas_types.htm

#### Pyrgus orcus

(Stoll, 1780)

http://eol.org/pages/183872/overview

http://butterfliesofamerica.com/L/pyrgus_orcus_types.htm

#### Siderone galanthis

(Cramer, 1775/76)

http://eol.org/pages/170707/overview

http://www.butterfliesofamerica.com/L/siderone_g_galanthis_types.htm

#### Siproeta stelenes

(Linnaus, 1758)

http://eol.org/pages/4068082/overview

http://butterfliesofamerica.com/siproeta_s_stelenes_types.htm

#### Smyrna blomfildia

(Fabricius, 1781)

http://eol.org/pages/164148/overview

http://www.butterfliesofamerica.com/L/smyrna_b_blomfildia_types.htm

#### Staphylus sp.

http://eol.org/pages/20450/overview

#### Taygetis acuta

(Weymer, 1911)

http://eol.org/pages/147615/overview

http://www.butterfliesofamerica.com/L/taygetis_acuta_types.htm

#### Taygetis laches

(Fabricius, 1793)

http://eol.org/pages/146471/overview

http://www.butterfliesofamerica.com/L/taygetis_l_laches_types.htm

#### Taygetis mermeria

(Cramer, 1779)

http://eol.org/pages/139915/overview

http://www.butterfliesofamerica.com/L/taygetis_m_mermeria_types.htm

#### Taygetis sylvia

(Bates, 1866)

http://eol.org/pages/146462/overview

http://www.butterfliesofamerica.com/L/taygetis_sylvia_types.htm

#### Temenis laothoe

(Cramer, 1779)

http://eol.org/pages/164154/overview

http://www.butterfliesofamerica.com/L/temenis_l_laothoe_types.htm

#### Telenassa teletusa

(Godart, 1823)

http://eol.org/pages/153045/overview

http://www.butterfliesofamerica.com/L/telenassa_t_teletusa_types.htm

#### Urbanus sp.

http://eol.org/pages/20632/overview

#### Yphthimoides straminea

(Butler, 1867)

http://eol.org/pages/40034120/overview

http://www.butterfliesofamerica.com/L/yphthimoides_straminea_types.htm

#### Zaretis isidora

(Cramer, 1779/80)

http://eol.org/pages/36076631/overview

http://www.butterfliesofamerica.com/L/zaretis_isidora_types.htm

#### Zaretis itys

(Cramer, 1777)

http://www.butterfliesofamerica.com/L/zaretis_isidora_types.htm

http://butterfliesofamerica.com/zaretis_i_itys_types.htm

## Discussion

The present study showed greater richness of species than the studies performed by Santana (2005), Rosa et al. (2011), Pedrotti et al. (2011), Favretto (2012), Ramos Soares et al. (2012). However, compared with records for several Brazilian states such as recorded by Isehard and Romanowski (2004), Marchiori and Romanowski (2006), Emery et al. (2006), Dessuy and Morais (2007), Pinheiro et al. (2008), Isehard et al. (2010), Ritter et al. (2011), Zacca and Bravo (2012), and Bogiani et al. (2012), the number of species found is lower. This fact could very well be explained by the sampled area size and sampling effort (Table 4).

In southeastern Brazil Mielke and Casagrande (1997) recorded 426 species in an area of ​​ 33,000 ha,for the Morro do Diabo State Park - São Paulo whereas the study by Brown Jr. and Freitas (2000a) in Santa Tereza – Espirito Santo, 297 species were registered in an area of ​​332,000 ha. In the state of Minas Gerais, Motta (2002) recorded 251 species in an area of ​​30 hectares in a region of Uberlândia; Silva et al. (2007) registred 91 species of butterflies in the PUC Minas Forest located in Belo Horizonte, in 7.0 ha; Ramos Soares et al. (2012) found 78 species in Americo Rene Giannetti Municipal Park with 0,018 ha.

The Nymphalidae was the family with greatest richness; this diversity can be explained by the fact that this family has great diversity in morphology and habits, as well as in environments with varying vegetation types (Brown Jr. and Freitas 1999) such as found in the Serra do Intendente State Park and the Peixe Tolo Natural Reserve.

In this study, the largest number of individuals collected (145) belongs to the subfamily Satyrinae. This family is important in analyses of disturbance studies (Devries and Walla 2001), in addition to being excellent predictors of the butterfly fauna of the Atlantic Forest (Brown Jr. and Freitas 2000b). From this subfamily, 12 individuals belonging to the *Taygetis laches* species were captured that has greater preference for more urbanly impacted environments (Silva et al. 2007).

*Eurema albula* and *Eurema elathea*, also registered in this site, have cosmopolitan habits and great adaptations for disturbed areas (Isehard et al. 2007, Bogiani et al. 2012). It is important to mention that *Morpho helenor*, which was well sampled – 51 individuals (Table 1), disappears quickly when severe disturbances and size reduction of forests occur (Santana 2005). These data demonstrate that the region could be severely impacted at some sites.

*Morpho helenor*, *Siproeta stelenes*, *Heliconius erato*, and *Heliconius ethilla* coincided with the study realized at the University Campus Darcy Ribeiro, in an urbanized area in the Federal District (Pinheiro et al. 2008). These are typical species of riparian areas, a characteristic of the sample site. *Euptychoides castrensis* is found in abundance in tropical rain forest environments, being registered in the states of São Paulo, Rio Grande do Sul, and Minas Gerais Pedrotti et al. (2011); this study obtained the same high record, a fact that corroborates with the presence of Atlantic Forest patches of vegetation in the studied sites.

There are no records of inventories for the Espinhaço mountain range within the state of Minas Gerais: this is the first published inventory for the region. This study and the only study in the Serra do Espinhaço about butterflies, conducted in Chapada Diamantina in Bahia - Brazil by Zacca and Bravo (2012) had similar predominance of species belonging to the family Nymphalidae. The species shared among these two studies are: *Adelpha pleasure*, *Archaeoprepona demophon*, *Ascia monuste*, *Callicore sorana*, *Colobura dirce*, *Dryas iulia*, *Eresia lansdorfi*, *Eryphanis reevesii*, *Eurema albula*, *Eurema elathea*, *Hamadryas amphinome*, *Hamadryas februa*, *Hamadryas feronia*, *Heliconius erato*, *Heliconius ethilla*, *Junonia evarete*, *Leptotes cassius*, *Pareuptychia ocirrhoe*, *Pyrgus orcus*, *Prepona laertes*, *Siproeta stelenes*, *Smyrna blomfildia*, *Taygetis laches*, *Temenis laothoe*, *Zaretis itys*, and *Yphthimoides straminea*.

It is emphasized that in this study the majority of butterflies species captured are typical of Cerrado and Atlantic Forest (Emery et al. 2006, Brown Jr. and Freitas 2000b).

Further investigation on biodiversity should be conducted and motivated in this region. The group of Lepidoptera showed great research and conservation potential for the Serra do Intendente State Park. The biodiversity information should be made available for decision makers, specially for regions such as the one studied, which is currently threatened by mining, tourism, and housing developments.

## Supplementary Material

XML Treatment for Adelpha pleasure

XML Treatment for Adelotypa malca

XML Treatment for Archaeoprepona demophon

XML Treatment for Autochton zarex

XML Treatment for Anartia amathea

XML Treatment for Ascia monuste

XML Treatment for Blepolenis batea

XML Treatment for Caligo arisbe

XML Treatment for Callicore sorana

XML Treatment for Catonephele acontius

XML Treatment for Colobura dirce

XML Treatment for Dryas iulia

XML Treatment for Euptychoides castrensis

XML Treatment for Eryphanis reevesii

XML Treatment for Eresia lansdorfi

XML Treatment for Eurema albula

XML Treatment for Eurema elathea

XML Treatment for Eurema phiale

XML Treatment for Eurema sp.

XML Treatment for Godartiana muscosa

XML Treatment for Hamadryas amphinome

XML Treatment for Hamadryas februa

XML Treatment for Hamadryas feronia

XML Treatment for Heliconius besckei

XML Treatment for Heliconius erato

XML Treatment for Heliconius ethilla

XML Treatment for Heliopetes omrina

XML Treatment for Junonia evarete

XML Treatment for Junonia genoveva

XML Treatment for Leptotes cassius

XML Treatment for Leptotes sp.

XML Treatment for Marpesia chiron

XML Treatment for Memphis moruus

XML Treatment for Memphis otrere

XML Treatment for Memphis ryphea

XML Treatment for Memphis sp.

XML Treatment for Morpho helenor

XML Treatment for Narope cyllarus

XML Treatment for Opsiphanes cassiae

XML Treatment for Opsiphanes quiteria

XML Treatment for Opoptera syme

XML Treatment for Pareuptychia ocirrhoe

XML Treatment for Paryphthimoides undulata

XML Treatment for Prepona laertes

XML Treatment for Pseudolycaena marsyas

XML Treatment for Pyrgus orcus

XML Treatment for Siderone galanthis

XML Treatment for Siproeta stelenes

XML Treatment for Smyrna blomfildia

XML Treatment for Staphylus sp.

XML Treatment for Taygetis acuta

XML Treatment for Taygetis laches

XML Treatment for Taygetis mermeria

XML Treatment for Taygetis sylvia

XML Treatment for Temenis laothoe

XML Treatment for Telenassa teletusa

XML Treatment for Urbanus sp.

XML Treatment for Yphthimoides straminea

XML Treatment for Zaretis isidora

XML Treatment for Zaretis itys

## Figures and Tables

**Table 1.** List of species collected in traps and active search in Serra do Intendente State Park, Minas Gerais, Brazil.

Species	Traps	Active Search
*Adelpha pleasure* (Hübner, 1823)8	1	0
*Adelotypa malca* (Shaus, 1902)	0	1
*Archaeoprepona demophon* (Linnaeus, 1758)	2	0
*Autochton zarex* (Hübner, 1818)	1	0
*Anartia amathea* (Linnaeus, 1758)	0	1
*Ascia monuste* (Linnaeus, 1764)	0	3
*Blepolenis batea* (Hübner, 1821)	1	1
*Caligo arisbe* Hübner, 1820	1	0
*Callicore sorana* (Godart, 1832)	3	0
*Catonephele acontius* (Linnaeus, 1771)	1	0
*Colobura dirce* (Linnaeus, 1764)	4	0
*Dryas iulia* (Fabricius, 1775)	0	2
*Euptychoides castrensis* (Shaus, 1902)	53	2
*Eryphanis reevesii* (Doubleday, 1849)	0	0
*Eresia lansdorfi* (Godart, 1819)	0	1
*Eurema albula* (Cramer, 1775)	0	3
*Eurema elathea* (Cramer, 1777)	0	2
*Eurema phiale* (Cramer, 1775)	0	6
*Eurema* sp.	0	1
*Godartiana muscosa* (Butler, 1870)	11	0
*Hamadryas amphinome* (Linnaeus, 1767)	6	0
*Hamadryas februa* (Hübner, 1816/24)	3	0
*Hamadryas feronia* (Linnaeus, 1758)	12	2
*Heliconius besckei* (E. Ménétriés, 1857)	3	1
*Heliconius erato* (Linnaeus, 1764)	1	1
*Heliconius ethilla* (Godart, 1819)	0	2
*Heliopetes omrina* (Butler, 1870)	0	1
*Junonia evarete* (Cramer, 1782)	0	1
*Junonia genoveva* (Cramer, 1782)	0	1
*Leptotes cassius* (Cramer, 1775)	0	2
*Leptotes* sp.	0	1
*Marpesia chiron* (Fabricius, 1775)	0	1
*Memphis moruus* (Fabricius, 1775)	12	0
*Memphis otrere* (Hübner, 1825)	1	0
*Memphis ryphea* (Geyer, 1834)	1	0
*Memphis* sp.	1	0
*Morpho helenor* (Cramer, 1782)	50	1
*Narope cyllarus* (Westwood, 1851)	3	0
*Opsiphanes cassiae* (Linnaeus, 1758)	2	0
*Opsiphanes quitera* (Stoll, 1782)	2	0
*Opoptera syme* (Hübner, 1822/26)	2	0
*Pareuptychia ocirrhoe* (Fabricius, 1777)	2	0
*Paryphthimoides undulata* (Butler, 1867)	1	0
*Prepona laertes* (Hübner, 1811)	1	0
*Pseudolycaena marsyas* (Linnaeus, 1758)	0	1
*Pyrgus orcus* (Stoll, 1780)	0	2
*Siderone galanthis* (Cramer, 1775/76)	1	0
*Siproeta stelenes* (Linnaeus, 1758)	0	1
*Smyrna blomfildia* (Fabricius, 1781)	2	0
*Staphylus* sp.	0	3
*Taygetis acuta* (Weymer, 1911)	1	0
*Taygetis laches* (Fabricius, 1793)	15	0
*Taygetis mermeria* (Cramer, 1779)	4	0
*Taygetis sylvia* (Bates, 1866)	1	0
*Temenis laothoe* (Cramer, 1779)	1	0
*Telenassa teletusa* (Godart, 1823)	0	16
*Urbanus* sp.	0	1
*Yphthimoides straminea* (Butler, 1867)	51	1
*Zaretis isidora* (Cramer, 1779/80)	5	0
*Zaretis itys* (Cramer, 1777)	1	0

**Table 2.** List of species collected during rainy seasons in Serra do Intendente State Park, Minas Gerais, Brazil.

Species	Traps	Active Search
*Adelpha pleasure* (Hübner, 1823)	1	0
*Adelotypa malca* (Shaus, 1902)	0	1
*Archaeoprepona demophon* (Linnaus, 1758)	1	0
*Autochton zarex* (Hübner, 1818)	1	0
*Anartia amathea* (Linnaeus, 1758)	0	0
*Ascia monuste* (Linnaeus, 1764)	0	0
*Blepolenis batea* (Hübner, 1821)	0	0
*Caligo arisbe* Hübner, 1820	3	0
*Callicore sorana* (Godart, 1832)	1	0
*Catonephele acontius* (Linnaeus, 1771)	1	0
*Colobura dirce* (Linnaeus, 1764)	4	0
*Dryas iulia* (Fabricius, 1775)	0	0
*Euptychoides castrensis* (Shaus, 1902)	25	0
*Eryphanis reevesii* (Doubleday, 1849)	0	0
*Eresia lansdorfi* (Godart, 1819)	0	0
*Eurema albula* (Cramer, 1775)	0	0
*Eurema elathea* (Cramer, 1777)	0	0
*Eurema phiale* (Cramer, 1775)	0	0
*Eurema* sp.	0	0
*Godartiana muscosa* (Butler, 1870)	9	0
*Hamadryas amphinome* (Linnaus, 1767)	6	0
*Hamadryas februa* (Hübner, 1816/24)	2	0
*Hamadryas feronia* (Linnaeus, 1758)	7	0
*Heliconius besckei* (E. Ménétriés, 1857)	1	0
*Heliconius erato* (Linnaeus, 1764)	0	0
*Heliconius ethilla* (Godart, 1819)	0	0
*Heliopetes omrina* (Butler, 1870)	0	0
*Junonia evarete* (Cramer, 1782)	0	0
*Junonia genoveva* (Cramer, 1782)	0	0
*Leptotes cassius* (Cramer, 1775)	0	2
*Leptotes* sp.	0	1
*Marpesia chiron* (Fabricius, 1775)	0	0
*Memphis moruus* (Fabricius, 1775)	6	0
*Memphis otrere* (Hübner, 1825)	0	0
*Memphis ryphea* (Geyer, 1834)	1	0
*Memphis* sp.	1	0
*Morpho helenor* (Cramer, 1782)	30	0
*Narope cyllarus* (Westwood, 1851)	1	0
*Opsiphanes cassiae* (Linnaeus, 1758)	2	0
*Opsiphanes quitera* (Stoll, 1782)	1	0
*Opoptera syme* (Hübner, 1822/26)	2	0
*Pareuptychia ocirrhoe* (Fabricius, 1777)	2	0
*Paryphthimoides undulata* (Butler, 1867)	0	0
*Prepona laertes* (Hübner, 1811)	0	0
*Pseudolycaena marsyas* (Linnaeus, 1758)	0	0
*Pyrgus orcus* (Stoll, 1780)	0	0
*Siderone galanthis* (Cramer, 1775/76)	0	0
*Siproeta stelenes* (Linnaeus, 1758)	0	0
*Smyrna blomfildia* (Fabricius, 1781)	2	0
*Staphylus* sp.	0	0
*Taygetis acuta* (Weymer, 1911)	1	0
*Taygetis laches* (Fabricius, 1793)	14	0
*Taygetis mermeria* (Cramer, 1779)	3	0
*Taygetis sylvia* (Bates, 1866)	0	0
*Temenis laothoe* (Cramer, 1779)	1	0
*Telenassa teletusa* (Godart, 1823)	0	0
*Urbanus* sp.	0	0
*Yphthimoides straminea* (Butler, 1867)	47	0
*Zaretis isidora* (Cramer, 1779/80)	1	0
*Zaretis itys* (Cramer, 1777)	0	0

**Table 3.** List of species collected during the dry seasons in Serra do Intendente State Park, Minas Gerais, Brazil

Species	Traps	Active Search
*Adelpha pleasure* (Hübner, 1823)	0	0
*Adelotypa malca* (Shaus, 1902)	0	0
*Archaeoprepona demophon* (Linnaus, 1758)	1	0
*Autochton zarex* (Hübner, 1818)	0	0
*Anartia amathea* (Linnaeus, 1758)	0	1
*Ascia monuste* (Linnaeus, 1764)	0	3
*Blepolenis batea* (Hübner, 1821)	1	1
*Caligo arisbe* Hübner, 1820	0	0
*Callicore sorana* (Godart, 1832)	0	0
*Catonephele acontius* (Linnaeus, 1771)	0	0
*Colobura dirce* (Linnaeus, 1764)	0	0
*Dryas iulia* (Fabricius, 1775)	0	2
*Euptychoides castrensis* (Shaus, 1902)	28	2
*Eryphanis reevesii* (Doubleday, 1849)	0	0
*Eresia lansdorfi* (Godart, 1819)	0	1
*Eurema albula* (Cramer, 1775)	0	3
*Eurema elathea* (Cramer, 1777)	0	2
*Eurema phiale* (Cramer, 1775)	0	6
*Eurema* sp.	0	1
*Godartiana muscosa* (Butler, 1870)	2	0
*Hamadryas amphinome* (Linnaeus, 1767)	0	0
*Hamadryas februa* (Hübner, 1816/24)	1	0
*Hamadryas feronia* (Linnaeus, 1758)	5	2
*Heliconius besckei* (E. Ménétriés, 1857)	2	1
*Heliconius erato* (Linnaeus, 1764)	1	1
*Heliconius ethilla* (Godart, 1819)	0	2
*Heliopetes omrina* (Butler, 1870)	0	1
*Junonia evarete* (Cramer, 1782)	0	1
*Junonia genoveva* (Cramer, 1782)	0	1
*Leptotes cassius* (Cramer, 1775)	0	0
*Leptotes* sp.	0	0
*Marpesia chiron* (Fabricius, 1775)	0	1
*Memphis moruus* (Fabricius, 1775)	6	0
*Memphis otrere* (Hübner, 1825)	1	0
*Memphis ryphea* (Geyer, 1834)	0	0
*Memphis* sp.	0	0
*Morpho helenor* (Cramer, 1782)	20	1
*Narope cyllarus* (Westwood, 1851)	2	0
*Opsiphanes cassiae* (Linnaeus, 1758)	0	0
*Opsiphanes quitera* (Stoll, 1782)	1	0
*Opoptera syme* (Hübner, 1822/26)	0	0
*Pareuptychia ocirrhoe* (Fabricius, 1777)	0	0
*Paryphthimoides undulata* (Butler, 1867)	1	0
*Prepona laertes* (Hübner, 1811)	1	0
*Pseudolycaena marsyas* (Linnaeus, 1758)	0	1
*Pyrgus orcus* (Stoll, 1780)	0	2
*Siderone galanthis* (Cramer, 1775/76)	1	0
*Siproeta stelenes* (Linnaeus, 1758)	0	1
*Smyrna blomfildia* (Fabricius, 1781)	0	0
*Staphylus* sp.	0	3
*Taygetis acuta* (Weymer, 1911)	0	0
*Taygetis laches* (Fabricius, 1793)	1	0
*Taygetis mermeria* (Cramer, 1779)	1	0
*Taygetis sylvia* (Bates, 1866)	1	0
*Temenis laothoe* (Cramer, 1779)	0	0
*Telenassa teletusa* (Godart, 1823)	0	16
*Urbanus* sp.	0	1
*Yphthimoides straminea* (Butler, 1867)	4	1
*Zaretis isidora* (Cramer, 1779/80)	4	0
*Zaretis itys* (Cramer, 1777)	4	0

**Table 4.** Comparison of Lepidoptera inventories with published checklists in Brazil

**Study**	**State (Brazil)**	**Biome**	**Sampled area**	**Richness**
Nery et al. 2014 (this study)	Minas Gerais	Caatinga,Cerrado and Atlantic Forest	13,447	60
Bogiani et al. 2012	Mato Grosso do Sul	Cerrado	60,5	62
Dolibaina et al. 2011	Paraná	Atlantic Forest	5,000	689
Silva et al. 2007	Minas Gerais	Cerrado	7	91
Bonfantti et al. 2011	Paraná	Atlantic Forest	27	166
Paluch et al. 2011	Pernabuco	Atlantic Forest	359	197
Mielke et al. 2008	Distrito Federal	Cerrado	Not applicable	335
Favretto 2012	Santa Catarina	Atlantic Forest	Not applicable	58
Marchiori and Romanowski 2006	Rio Grande do Sul	Steppe Savanna	1,617.14	97
Emery et al. 2006	Distrito Federal	Cerrado	Notpplicable	504
Pinheiro et al. 2008	Distrito Federal	Cerrado	50,5	128
Mielke and Casagrande 1997	São Paulo	Atlantic Forest	33,845	426
Silva et al. 2012	Minas Gerais	Cerrado and Atlantic Forest	151	45
Isehard et al. 2007	Rio Grande do Sul	Atlantic Forest	1,606.60	149
Santana 2005	Mato Grosso	Cerrado and Atlantic Forest	480.02	69
Ramos Soares et al. 2012	Minas Gerais	Atlantic Forest	18.2	78
Isehard et al. 2010	Rio Grande do Sul	Atlantic Forest	1,606.60	277
Isehard and Romanowski 2004	Rio Grande do Sul	Atlantic Forest	54,600	292
Pinheiro and Emery 2006	Distrito Federal	Cerrado	25,000	507
Brown Jr. and Freitas 2000a	Espirito Santo	Atlantic Forest	Not applicable	297
Motta 2002	Minas Gerais	Cerrado	30	251
Silva et al. 2010	Minas Gerais	Atlantic Forest	36,970	83
